# Structural Insights Reveal the Dynamics of the Repeating r(CAG) Transcript Found in Huntington’s Disease (HD) and Spinocerebellar Ataxias (SCAs)

**DOI:** 10.1371/journal.pone.0131788

**Published:** 2015-07-06

**Authors:** Arpita Tawani, Amit Kumar

**Affiliations:** Centre for Biosciences and Biomedical Engineering, Indian Institute of Technology Indore, Indore, Madhya Pradesh, India; Bioinformatics Institute, SINGAPORE

## Abstract

In humans, neurodegenerative disorders such as Huntington’s disease (HD) and many spinocerebellar ataxias (SCAs) have been found to be associated with CAG trinucleotide repeat expansion. An important RNA-mediated mechanism that causes these diseases involves the binding of the splicing regulator protein MBNL1 (Muscleblind-like 1 protein) to expanded r(C**A**G) repeats. Moreover, mutant huntingtin protein translated from expanded r(C**A**G) also yields toxic effects. To discern the role of mutant RNA in these diseases, it is essential to gather information about its structure. Detailed insight into the different structures and conformations adopted by these mutant transcripts is vital for developing therapeutics targeting them. Here, we report the crystal structure of an RNA model with a r(C**A**G) motif, which is complemented by an NMR-based solution structure obtained from restrained Molecular Dynamics (rMD) simulation studies. Crystal structure data of the RNA model resolved at 2.3 Å reveals non-canonical pairing of adenine in 5´-CAG/3´-GAC motif samples in different *syn* and *anti* conformations. The overall RNA structure has helical parameters intermediate to the A- and B-forms of nucleic acids due to the global widening of major grooves and base-pair preferences near internal AA loops. The comprehension of structural behaviour by studying the spectral features and the dynamics also supports the flexible nature of the r(C**A**G) motif.

## Introduction

RNA plays an essential role in normal and abnormal cell functions, which makes it a crucial target for the selective binding of small molecules [1]. Due to a lack of understanding of the RNA motifs that bind or become recognised by small molecules, many RNA targets are not exploited [2]. Triplet repeat-containing transcripts may be one such type of target [3]. These repeats undergo pathogenic expansion, which causes non-curable triplet-repeat expansion diseases (TREDs) [4]. More than 15 neurological diseases are known to be caused by the expansion of these trinucleotide repeats, and the majority of a group of diseases are caused by the expansion of CAG repeats [5]. These CAG repeats may be located in the protein-coding region of corresponding genes or untranslated regions (UTRs). Disorders caused by mutant proteins include Huntington’s disease (HD), dentatorubral-palidoluysian atrophy (DRPLA), spinal and bulbar atrophy (SBMA) and most of the spinocerebellar ataxias (SCAs) 1,2,3,6,7,17 [6–8]. Indeed, CAG codes for glutamine (Q) during translation, but due to mutation, expanded CAG repeats are translated into polyglutamine (polyQ) tracts. These polyQ tracts get incorporated into mutant proteins, thus categorising these disorders as polyglutamine (polyQ) diseases. The pathogenesis caused by these mutant proteins have various mechanisms including gain-of-toxic function [9], normal protein function may increase to a toxic level [10], aggregate formation [11] and the sequestration of other proteins such as CREB-binding protein (CBP) [12] and polyglutamine tract-binding domain protein (PQBP-1) [13].

In addition to the CAG repeats located in coding regions, expansion in UTRs also causes pathogenesis [14–16]. Such disorders include SCA12, which occurs due to the presence of a CAG repeat motif in the 5´UTR of the PPP2R2B gene [5], and likely SCA8 [17] and SCA10 [18]. Mutant transcript-mediated toxicity is due to the sequestration of regulatory proteins and factors by CAG repeat motifs e.g., muscleblind-like1 (MBNL1), a splicing regulator protein [19], nucleolin, a nucleolar protein [20,21] and many other transcription factors [22]. The recruitment of these proteins and factors to expanded CAG repeat motifs results in the obstruction of many biological pathways such as reduction in cellular rRNA, dysregulated gene transcription, and the assembly of aberrant short silencing RNAs [23]. Such studies may potentially provide a strategy for treating disorders. Previous studies have focused on reductions in mutant protein as a therapeutic approach, such as intrabodies and artificial polypeptides, which target the polyQ domain [24]. However, recent studies have shown that obstruction in pathogenesis can also be achieved by the binding of small molecules to r(C**A**G) repeats that corrects splicing defects in disorders caused by RNA gain-of-function [25]. These small molecules have been identified using virtual screening or designer modular assembly strategies [25,26]. These small molecules recognise the structure of RNA rather than its sequence, which makes this strategy more advantageous than other therapeutic approaches [26].

Over the past decade, the structural characteristics of RNAs containing CAG repeat motifs were explored by biochemical methods [27–31]. These studies have shown that the long CAG repeat tracts form stable hairpins in which the C-G and G-C bases form closing pairs, and the A-A pairs interact within hairpins by forming 1x1 nucleotide internal loops [13,31]. Better resolved information about the structures can be acquired by NMR spectroscopy and X-ray crystallography experiments [2,8,32–34], and such studies also show stable hairpin formation by long CAG repeat tracts [31,35]. In one study, the crystal structure of an RNA model resolved at 0.95 Å revealed A-A wobble pairing in 5´ r(GGCAGCAGCC)_2_. This A-A interaction causes local unwinding of RNA duplexes in which adenosines adopt an *anti* conformation with a single H-bond between the C2H2 and N1 of paired adenosines [8]. Similarly, A-A pairing is also observed in a ribosome model (PDB code 1FFK) with adenosines in the *anti* conformation and the N6 amino group in an H-bond with N1 [36].

However, neither of these studies reveal the dynamic behaviour of the adenosines, which is likely to be observed in motions involving large amplitude changes. In contrast, another group reported the crystal structure of self-complimentary duplex RNA containing CAG repeats resolved at 1.65 Å resolution. This study revealed the dynamic behaviour of AA internal loops in 5´ r(UUGGGC(C**A**G)_3_GUCC)_2,_ with these loops favouring the *anti-anti* conformation. However, simulation studies have shown that these 1x1 AA nucleotide internal loops are stable in the *syn-anti* conformation. Furthermore, this conformation is affected by the binding of ions or small molecules to 1x1 AA nucleotide internal loops [6].

The functions of RNA models can be explained by examining the dynamics of their systems, which further compliment static structural data [37,38]. In biomolecules, molecular motions occur on a time scale ranging from femtoseconds to seconds. NMR data provide information on the fast dynamic motions in the sub-nanosecond time range. Better insight into the binding of small molecules or other factors may be gained by understanding the behaviour of such motions [39].

It is clear that mutant transcripts play an essential role in the pathogenesis of CAG repeat disorders. Thus, to explore the role of RNA structures in pathogenesis, it is necessary to obtain knowledge of conformational flexibility and dynamic behaviour. Here, we report the crystal structure of an RNA model containing three consecutive 5´-CAG/3´-GAC motif, which is complemented by an NMR-based solution structure and restrained Molecular Dynamics (rMD) simulation studies.

## Materials and Methods

### Purification of RNA

The CAG motif containing RNA 5´ r(UUGGGCC**A**GC**A**GC**A**GGUCC)_2_ was purchased from Integrated DNA Technologies, Inc. in a desalted and deprotected form. Purification of the RNA was performed using a Waters HPLC instrument with an attached UV-Vis detector. The RNA was suspended in water and passed through an XTerra Prep MS C18 HPLC column (7.8 mm × 150 mm, 5 μm). Elution was performed by applying a linear gradient (from 100 to 0% 10 mM triethylammonium acetate (pH = 7) in acetonitrile over 55 minutes) with a flow rate of 2 ml/min (t_R_ = 25 mins). Subsequent desalting was performed by a Sephadex PD-10 prepacked size-exclusion column. The final concentration of the RNA duplex was calculated from its absorbance at 260 nm (at 95°C). Hyther Server, based on nearest neighbour thermodynamics, was used to determine the molar extinction coefficients [40,41].

### Crystallisation Method

RNA was dissolved in DEPC-treated water to a final concentration of 1.2 mM, and it was annealed by heating to 60°C followed by subsequent cooling to room temperature. The Qiagen Nucleix Suite kit was used to screen and optimise the crystallisation conditions yielding crystals that diffract well. High-quality crystals were obtained in 50 mM Tris HCl (pH 8.5), 25 mM magnesium sulphate and 1.8 M ammonium sulfate using the sitting drop vapour diffusion method within 2–3 days at 18°C.

### Data Collection and Structure Refinement

A complete diffraction dataset at 2.3 Å resolution was collected using liquid nitrogen-immersed flash-frozen crystals. ADSC CCD detectors on beamline 9–1 at SSRL or beamline LS-CAT (21-ID) at the Advanced Photo Source (Argonne National Laboratory, Argonne, IL) under cryoconditions (100 K) were used to collect the data. The data were scaled and integrated using HKL2000 [42]. Initial phases for the crystal were determined by molecular replacement in PHASER [43], a module in PHENIX interfaces [44]. A 19-bp standard form of RNA generated from COOT [45] served as a phasing model. COOT was used for multiple rounds of manual fitting and rebuilding of the structure. The structure was further refined in the CCP4 program suite [46]. Data collection, processing and refinement statistics are listed in S1 Table.

### Calculation of Electrostatic potential and structural parameters

Electrostatic potential was calculated from the corresponding crystal structure model. The non-linear Poisson Boltzmann equation [47] was used to solve the electrostatic surface potential. Hydrogen atoms were assigned and positioned on the RNAs in a manner in which they did not pose steric conflict. AMBER was used to assign partial atomic charges and atomic radii based on Amber99 force field [48]. ABPS was employed to determine the surface electrostatic potential of the RNA models [49]. The RNA molecule was considered as a low dielectric medium within the volume enclosed by its solvent-accessible surface (probe radius = 1.4 Å). Electronic polarisability effects were accounted for by employing a dielectric constant of 2 while considering the surrounding solvent as a continuum with a dielectric constant of 80. The 2.0 Å ion-exclusion radius was added to account for the ion size on the surface of the RNA molecules. Molecular surfaces were constructed using ten grid points per square angstrom. A sequential focusing method [50] was used to calculate the electrostatic surface potential. These electrostatic potential surfaces represent solvent excluded molecular surfaces. Initially, a coarse grid was exploited to solve the equation. Subsequently, the equation was refined using Dirichlet boundary conditions to obtain a finer grid [51]. The electrostatic calculations were performed at 298 K.

3DNA, a software package, was used to calculate helical parameters, torsional angles and groove widths [52]. Vector-connecting C1’ atoms were used for sequence-independent measurements to rule out computational artefacts arising from non-canonical base pairing.

### Nuclear Magnetic Resonance (NMR)

RNA duplexes with a single CAG motif, 5´ r(CCGC**A**GCGG)_2,_ and three CAG motifs, 5´ r(GGGCC**A**GC**A**GC**A**GGUCC)_2_, were used for NMR spectroscopy. RNA samples were prepared by resuspending lyophilised RNA in 10 mM phosphate buffer, pH 7.2, 0.1 M NaCl, and 50 mM EDTA in 10% D_2_O. NMR spectra were recorded with a Bruker NMR spectrometer with field strengths of 400 and 500 MHz. One dimensional proton spectra were recorded at various temperatures. Spectral assignments were performed using NOESY (Nuclear Overhauser Effect SpectroscopY), DQF-COSY (Double Quantum Filtered Correlation SpectroscopY) and TOCSY (Total Correlation SpectroscopY).

Two-dimensional (2D) NOESY was performed acquiring 4096 data points with 64 transients for each of the 296 FID signals. The spectra were recorded at 288, 298 and 308 K with mixing times of 300, 200 and 100 ms. A NOESY spectrum was recorded using an excitation exculpating pulse sequence to suppress residual water signals.

SPARKY was used to visualise the spectra and calculate ^1^H-^1^H NOE distances. These distances were used to restrain the RNA duplex for restrained Molecular Dynamic simulation studies.

The minimisation and restrained Molecular Dynamics (rMD) simulation were performed using Discovery Studio 3.5 (Accelerys Inc., USA). The RNA duplex was built using the Macromolecule module in Discovery Studio 3.5. After typing the duplex with a CHARMM force field [53], the model was solvated with an explicit solvent model such that the nucleic acid atoms were surrounded by an orthorhombic periodic box of TIP3P [54] water molecules extending to 20 Å. To effectively consider the long-range electrostatic interactions, minimisation was performed by the Particle Mesh Ewald (PME) method [55]. A total of 1000 steps using the steepest descent minimisation were performed using SHAKE [56] with bonds containing hydrogen.

After minimisation and optimisation, the RNA duplex was subjected to Standard Dynamic Cascade. Within this cascade, the system was again minimised with a steepest descent minimisation of 500 steps followed by a conjugate gradient minimisation of 500 steps. The system was then subjected to gradual heating from 50 to 300 K for 4 picoseconds. The molecule was equilibrated for 10 picoseconds with a time step of 2 femtoseconds at a constant temperature of 300 K. Equilibration was followed by production runs under a constant temperature (300 K) with PME treatment for electrostatic interactions, SHAKE for bonds containing hydrogen and a 2 femtosecond time step. Simulations were performed for 25 nanoseconds, and 100 conformations were generated. The output files were analysed with Discovery Studio 3.5 based on properties such as potential energy of the system, root mean square deviation (RMSD) and the presence of hydrogen bonds.

## Results and Discussion

### Crystal Structure of 3x2 CAG motifs containing RNA show the dynamics of AA internal loops

Here, we report the crystal structure of RNA duplexes containing three consecutive 5´-CAG/3´-GAC motifs showing dynamics, which is further validated by NMR studies. The duplex crystallised in the double-stranded helical structure and the structure was refined to resolution of 2.3 Å.

**Fig 1:**
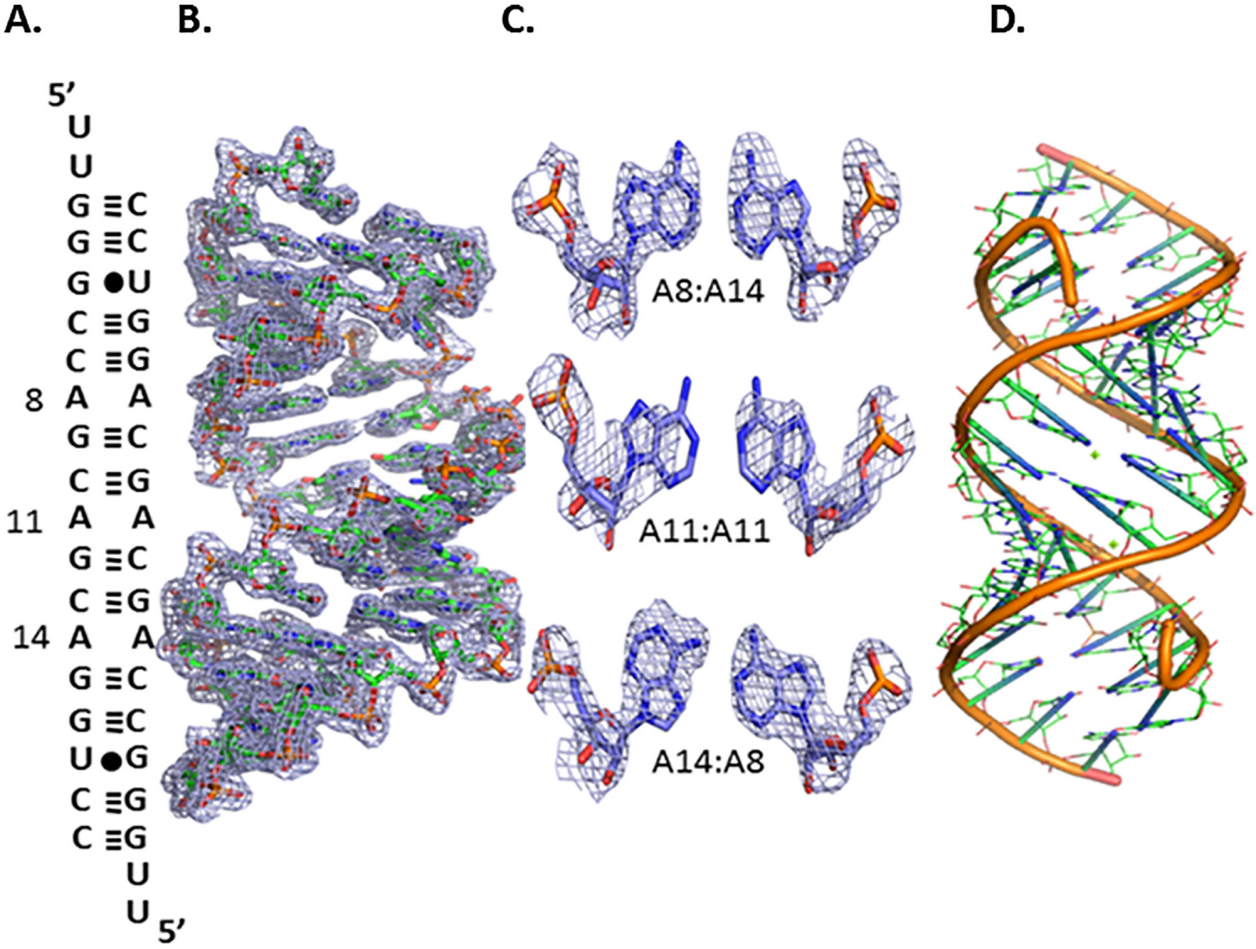
The secondary structure and refined structure of the RNA construct 5´ r(UUGGGCCAGCAGCAGGUCC)_2_. A. The secondary structure of oligonucleotide r(CAG) repeat duplex model that allowed crystal growth. B. The global structure of the RNA including the electron density map at 1.0 σ C. The electron density map of non-canonical A-A pairs at 1.0σ. D. The backbone structure of the RNA construct.

The RNA duplex was constructed to contain 5´ UU dangling ends and a duplex region flanking the three CAG motifs. The duplex region adjacent to the 5´-CAG/3´-GAC motifs imparts stability to the duplex and may be used for phasing (Fig 1A). These regions non-covalently bind to heavy atoms and heavy atom derivatives, which were used to infer the phases lost during data collection [50]. Electron density maps contoured at 1.0 σ for the AA internal loops were in accordance with different conformations of the internal AA loops (Fig 1B). The central AA internal loop and one of the terminal 1x1 nucleotide AA internal loops have both adenines in an *anti* conformation. Despite being in the *anti* conformation, one of the A's in the central AA internal loop was slightly tilted and did not have well-defined electron density (Fig 1C). This observation stipulates a dynamic nature for the AA internal loop. In addition, the third AA internal loop had one of the A’s in the *syn* conformation with the other in the *anti* conformation.

Previously published crystallographic studies revealed that RNA duplexes with two 5´-CAG/3´-GAC motifs fit remarkably well within regular A-helices, and both adenosines are in the *anti* conformation with a single hydrogen bond [8]. Furthermore, another report based on an X-ray crystal structure and MD simulations suggested that adenosines can adopt the *syn*-*anti* conformations favoured by Na^+^ ions although the global minimum conformation is *anti*-*anti* [6]. However, the X-ray crystal results here show that the *syn*-*anti* conformational change in the adenines was independent of Na^+^ ions. Moreover, the existence of 5´-CAG/3´-GAC motifs in different conformations reveal that the repeats can be in multiple conformations, and the AA pairs fits well in the helix with the backbone remaining intact despite of the dynamic nature of the AA internal loop (Fig 1D).

**Fig 2:**
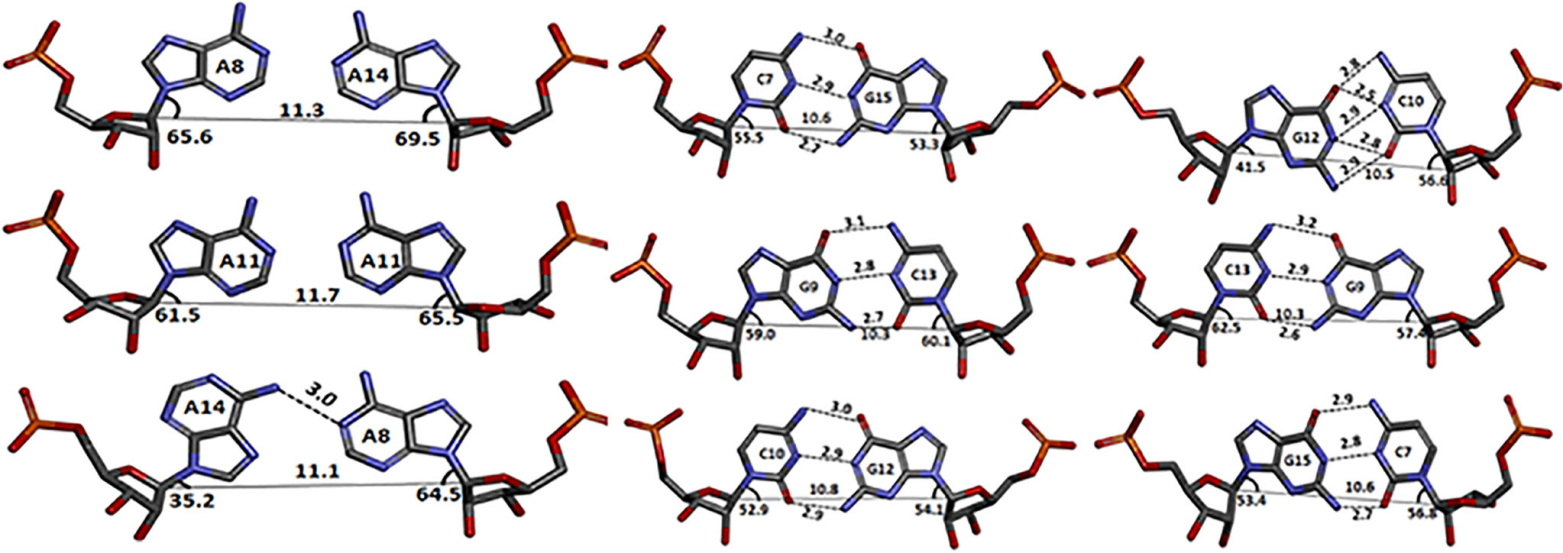
Three dimensional structure of the 1x1 nucleotide AA internal loop and its closing base pairs for RNA construct 5´ r(UUGGGCCAGCAGCAGGUCC)_2_. Each of the loop closing pairs has geometry consistent with that of Watson-Crick GC base pairs. The distance values (in Å) are labeled for hydrogen bonds (dashed lines); the C1´-C1´ distances (solid lines).

More interestingly, the 1x1 nucleotide AA internal loops show different pairing geometries (Fig 2). Two of the three 1x1 nucleotide AA internal loops have a zero hydrogen bond conformation, while the remaining loop has a single hydrogen bond conformation. In one hydrogen bond geometry, the hydrogen bond is between the hydrogen atom of the exocyclic amine (N6) of A14 and the N1 atom of A8. Zero hydrogen bond geometry has a distance greater than standard hydrogen bonds. The 1x1 nucleotide AA internal loops in the structure samples between zero and one hydrogen bond and do not disturb the loop-closing base pairs, which is evidence of their dynamic nature. Thus, this dynamic structure of the 1x1 internal AA loop supports a model for expanded CAG repeats that exhibit multiple conformations.

The 3DNA software package was used to analyse the comprehensive helical framework of the RNA. Sequence-independent helical parameters based on interstrand vectors connecting the C1´ atoms of paired residues were used to measure the helix properties. The C1´-C1´ distances between the CG closing pairs averaged 10.5 Å (Fig 2), whereas the distance between the AA pairs is greater than the standard distance of 10.5 Å. This observation explains the global widening of the major grooves of RNA as purine-purine pairs increases C1´-C1´ distances to 12.7 Å (S4 and S5 Tables).

Unlike a previous study reporting that intramolecular hydrogen bonding drives the 5´ UU dangling ends into the major groove of RNA, which increases the width of the groove [2], groove widening is actually associated with the orientation of the AAs in RNA duplexes as evident by the solution structure of RNA constructs with single C**A**G motifs without dangling UU ends (S5 Table).

**Fig 3:**
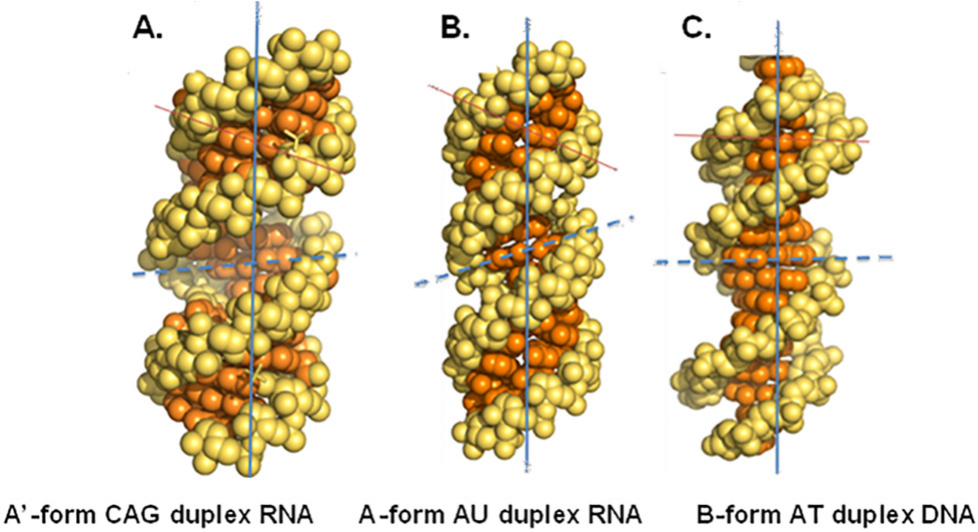
Ball and stick model of nucleic acid. Ball and stick model of various types of nucleic acid helical forms, showing base inclination angle axis (solid red line); diameter of groove (dashed blue line).

When the refined structure of the RNA duplex was compared with a construct in which the 1x1 AA internal nucleotides were replaced with AU pairs (A-form) and a B-form DNA duplex, the inclination angles of the bases were found to be lower compared with the A-form of RNA. Such changes in the inclination of bases could be derived from stacking purine interactions [51]. Thus, a RNA duplex containing a CAG motif was found to have a conformation (A´-form) intermediate to the A- and B- forms of the nucleic acid structure (Fig 3). Such A´-forms of RNA are characterised by lower inclination angles in comparison with the A-form and a widened major groove as evident by CGG motif repeats in RNA, which causes Fragile X-syndrome [57–59]. This widening of the major groove occurs due to the presence of AA pairs, which makes it more accessible to binding proteins or small molecules. Moreover, this A´-helical form could provide unique binding sites for protein or small-molecule ligands over DNA or RNA duplexes.

**Fig 4:**
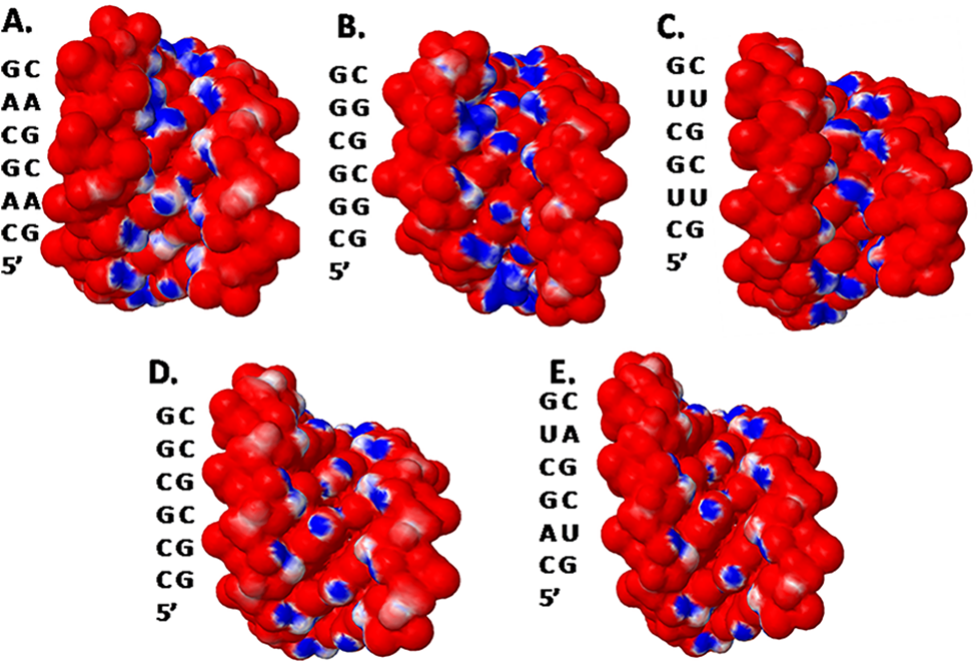
Comparison of the electrostatic charge distributions for CAG structure to duplex RNA and DNA. Panels A-D are electrostatic charge distributions; **A.** CAG structure; **B.** CGG structure (PDB 3SJ2); **C.** CUG structure (PDB 3SZX) and; **D.**, **E.** a structure in which the 1x1 nucleotide AA internal loops in the CAG construct were replaced with GC pairs and AU pairs, respectively.

Furthermore, this refined structure also yields interesting features about the electrostatic differences between canonically paired RNA duplexes and r(C**A**G) repeat RNAs. The electrostatic distribution shows that these repeats have a larger density of partial positive charges in the minor groove (Fig 4A–4E), which may be exploited for the binding of small molecules.

### NMR results for 1x2 CAG and 3x2 CAG motifs containing RNA showing the dynamics of AA internal loops

**Fig 5:**
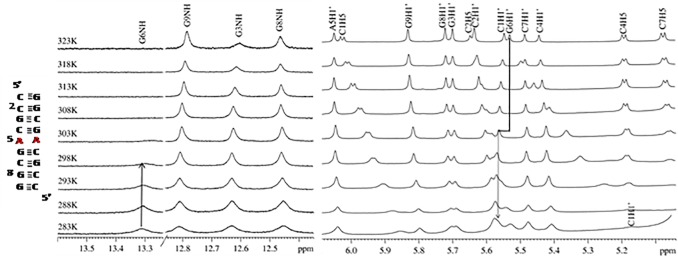
Temperature dependent ^1^H NMR spectra for 5´ r(CCGCAGCGG)_2_ showing imino region and sugar 1´ proton region. The upfield shift of G6H1´ resonance (left), downfield shift of A5H1´ resonance (right) and a rapid downfield shift of C1H1´ resonance (right) is clearly seen.

To validate the dynamic nature of the non-canonical adenine pairs observed by crystal structure, NMR spectroscopy was used as it allows structural determination in aqueous environments near physiological conditions. One-dimensional proton NMR spectra was recorded for 5´ r(CCG**C** **A** **G**CGG)_2_ at various temperatures. With an increase in temperature, the broadening of proton resonance peaks in the imino region (G6NH) was observed (Fig 5). The peak broadened with a gradual decay as the temperature was increased from 283 to 293 K, and it eventually disappeared at room temperature. Such a spectrum represents the fast exchange of G6NH with solvent as the imino proton becomes accessible to the solvent due to base flipping, which indicates the dynamic nature of the non-canonical adenine pair (A5) near guanine. The existence of multiple adenine conformations was reinforced by the A5H1´ resonance peak in the sugar 1´ proton region. With an increase in temperature, a slight downfield shift in A5H1´ resonance and upfield shift in G6H1´ resonance further corroborated evidence for the dynamics of adenine pairs. In addition, terminal C1H1´ resonance showed a rapid downfield shift due to the rapid exchange of exposed protons with solvent (Fig 5).

To support the above observations, NMR experiments were also performed using RNA constructs containing 3x2 CAG motifs (S1 Fig). The conformational flipping of adenine might lead to some perturbation in the hydrogen bonds of neighbouring GC pairs or increased stacking interactions between the guanine base and sugar. This may be evident by the appearance of an additional G13 imino signal in the 3x2 CAG RNA sequence (S1 and S2 Figs). The appearance of additional resonances for G13NH and G14NH indicates the likely dynamics arising due to a transition between two different conformations for the neighbouring adenine. However, due to the overlapping of the resonance peaks of the sugar, base and imino resonances (S3 and S4 Figs) (e.g., G7NH/G10NH), a simple RNA construct containing 1x2 CAG motifs was used in further studies.

**Fig 6:**
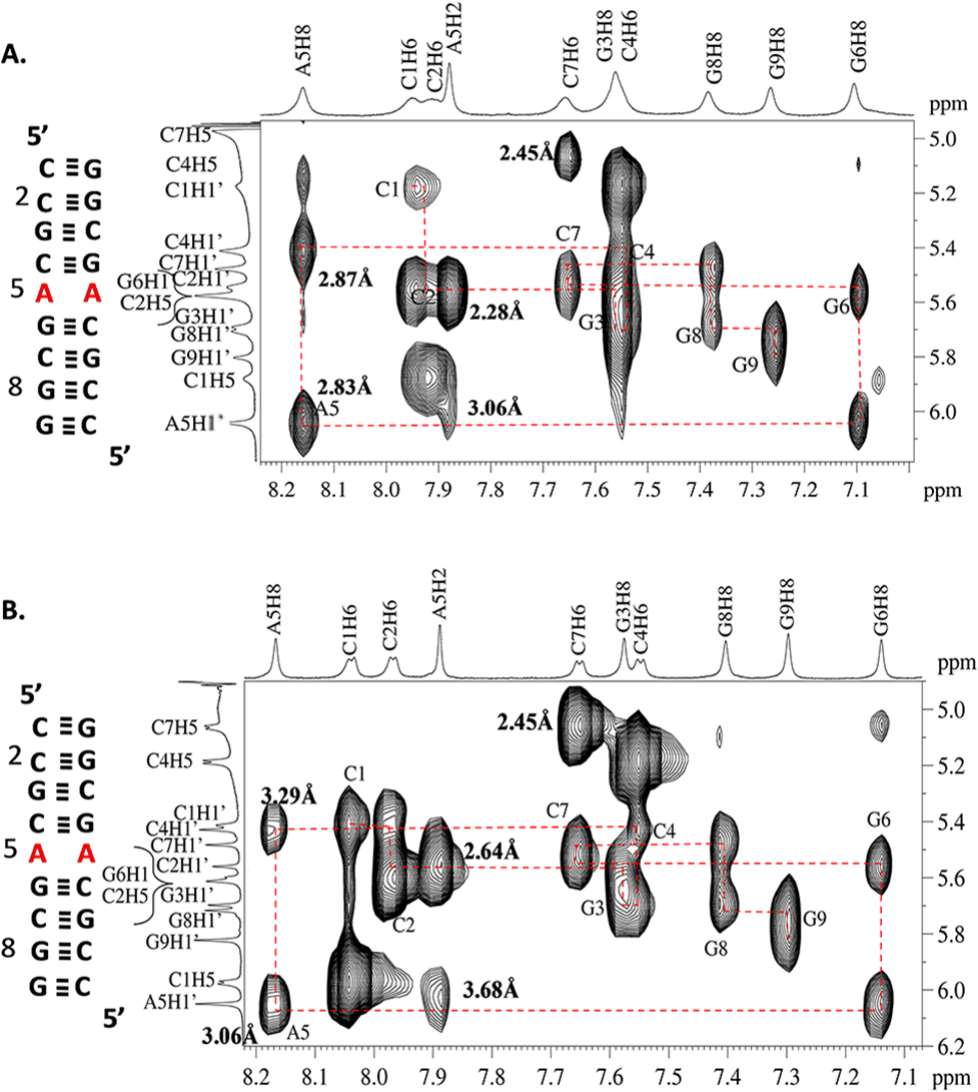
Portion of 300ms NOESY spectrum showing base to 1´ region for the sequence for 5´ r(CCGCAGCGG)_2_ at different temperatures A. 288 K and B. 308 K. The distances marked in the spectra shows the perturbation owing to change in temperature.

NOESY walk examines the sequential connectivity to sugars and succeeding base protons [60]. The sequential assignment for the 5´ r(CCGC**A**GCGG)_2_ duplex in NOESY spectra collected at 300 ms mixing time at two different temperatures, 288 and 308 K, show that the interproton distance between A5H8 and G6H8 increases with increasing temperature. This result indicates the existence of adenine conformational dynamics. In addition, the A5H1´- A5H2 cross peak intensity also varied with a change in temperature (Fig 6A and 6B). This change in intranucleotide (A5) resonance peak intensity also indicates the probability of adenine dynamics. Similarly, disappearance of the cross peak between C4H5 and A5H8 at 308 K further supports the above results (Fig 6B).

### Solution structure of 1x2 CAG motifs containing RNA as revealed by restrained Molecular Dynamic (rMD) simulation studies

To explicitly examine the conformational flexibility and dynamic nature of AA pairs, rMD simulations were performed using a RNA construct containing a single CAG motif, 5´ r(CCG**C** **A** **G**CGG)_2_, based on distances obtained from NOESY experiments. The duplex was restrained based on the ^1^H-^1^H NOE distances and Watson-Crick base pairing. The duplex was minimised to the lowest energy conformation and simulated using restrained Molecular Dynamics. One hundred distinct structures were generated in a 25 nanosecond simulation run.

**Fig 7:**
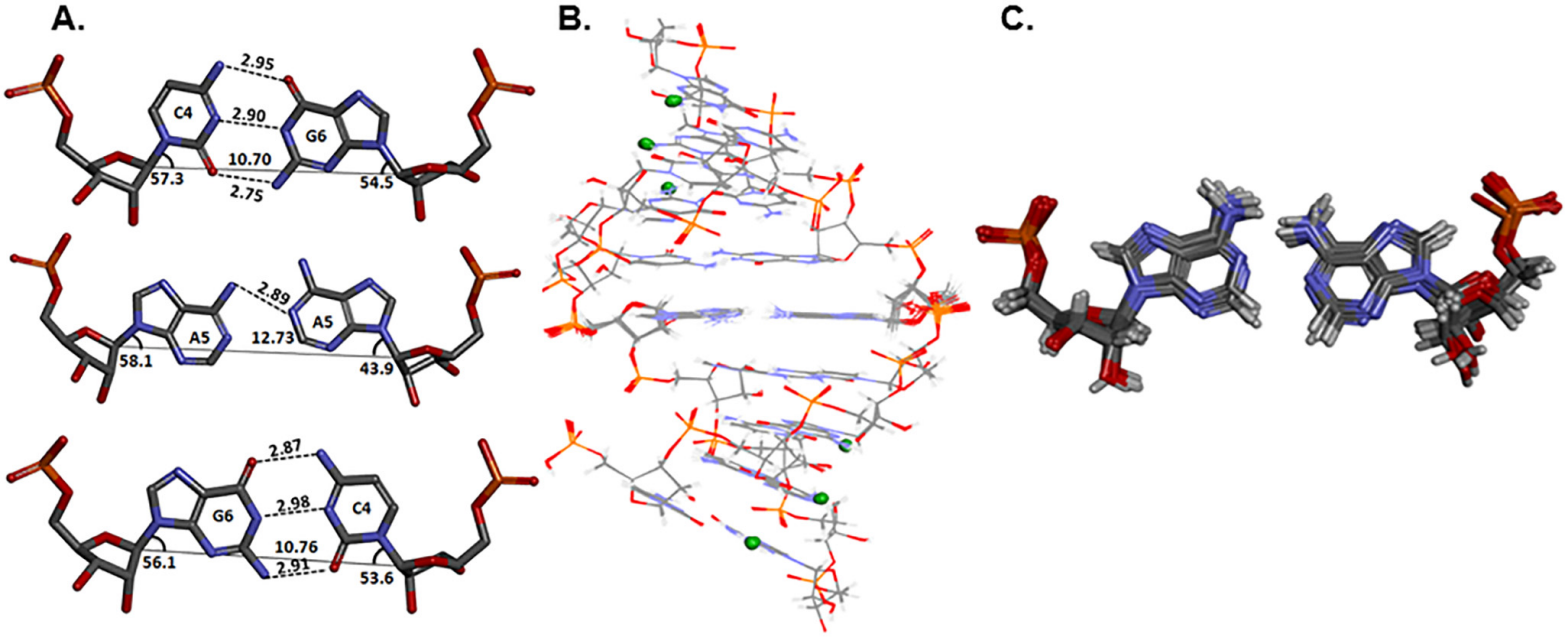
Lowest energy conformation of 5´ r(CCGCAGCGG)_2._ A. The lowest energy conformation of CAG motif obtained after rMD simulation of 5´ r(CCGCAGCGG)_**2**_. B. Ensemble of ten lowest energy structures of 5´ r(CCGCAGCGG)_**2**_ obtained after rMD simulation. C. Ensemble of ten lowest energy structures of AA pairs of 5´ r(CCGCAGCGG)_**2**_ obtained after rMD simulation.

Analysis of the AA pair geometry showed the existence of zero and one hydrogen bonds in the simulation trajectory, which is analogous to the crystal structure. The lowest energy conformation observed after simulation showed a single hydrogen bond between the non-canonical adenine pair (Fig 7A). In contrast with the AA pair, the loop closing GC pairs had a geometry consistent with that of Watson-Crick GC base pairing (Fig 7A). Furthermore, the C1´-C1´ distances between the adenine pair and loop-closing GC pairs were in line with the crystal data, thus supporting the model for an A´- form of RNA [57] (Fig 7A).

## Conclusion

In summary, we studied RNA models containing one and three 5´-CAG/3´-GAC motifs and such 5´-CAG/3´-GAC repeats beyond a limit known to cause Huntington’s disease. We have utilised the same RNA construct as used by Yildirim *et al*. [6] for crystallization and observed similar kind of dynamics in 1x1 AA nucleotide internal loops with *anti-anti* and *syn-anti* conformations. Additionally, we have also performed NMR experiments for both of these RNA constructs to observe the conformational dynamics in solution. Analysis by X-ray crystal structure, NMR spectroscopy and rMD simulations reveal that the 5´-CAG/3´-GAC motif is dynamic in nature. The likely dynamics of these motifs are due to the hydrogen bonding pattern and stacking interactions of non-canonical adenine pairs. This refined structure yielded interesting features that provide information about electrostatic distribution and the A'-helical form of nucleic acids that may provide unique binding sites for proteins or small-molecule ligands over canonically paired duplex DNA or RNA. The X-ray crystal results are in accordance with previous studies showing the *syn-anti* conformation of adenines that is independent of Na^+^ ions. Unlike the previous report of Yildirim *et al*., two *anti-anti* and one *syn-anti* conformation were observed for three 1x1 AA nucleotide internal loops. This may be due to differences in the buffer conditions used for crystal growth. As in crystallography, the multiple conformations were ‘snapshots’ of a dynamic system, so, it was very difficult to observe particular transitions between the different conformations of a dynamic system. These could be correlated to the observed differences in the conformation of the 1x1 AA nucleotide internal loops with that of Yildirim *et al*. report. Further, our crystal structure shows A'- form RNA due to the effect of base inclination and widening of major groove in CAG motif. Similar A'-form were also observed in CCG RNA structure in which 5´-CGG/3´-GGC motif also forms *syn-anti* conformation [58]. Furthermore, previous theoretical studies by Yildirim *et al*. [6] show that *anti*-*anti* structure is the most favoured and stable state, but we never achieved the *syn* conformation within the NMR time scale during our NMR experimental studies, however we observed dynamics in 5´-CAG/3´-GAC motif in both RNA constructs used in the present study.

Thus, deducing this RNA structural information will aid in enhancing the understanding of diverse RNA motifs, which may prove essential for therapeutic development. These studies will facilitate our progress toward deciphering the recognition of RNA by small molecules to target HD and SCAs. Understanding the structural and dynamic characteristics of RNA will directly influence our potential to eventually regulate cell function at the RNA level and promote new approaches for combating diseases by specifically targeting RNA or RNA-protein interactions, thus advancing RNA as a drug target.

### Accession number

The atomic coordinates and structural factors have been deposited in Protein Data Bank under the accession code 4YN6 and 2MS5.

## Supporting Information

S1 Fig Temperature dependent 1H NMR spectra for 5´ r(UUGGGC(CAG)3GUCC)2 showing imino region. One dimensional proton spectra for 5´ r(UUGGGC(C**A**G)_3_GUCC)_2_ showing imino proton region at variable temperature. Asterisk mark attributes to the appearance of new resonance due to the dynamics in adenine pairs.
(TIF)

S2 Fig Temperature dependent 1H NMR spectra for 5´ r(UUGGGC(CAG)3GUCC)2 showing base and sugar 1´ proton region. One dimensional proton spectra for 5´ r(UUGGGC(C**A**G)_3_GUCC)_2_ showing base and sugar 1**´** region at variable temperature.
(TIF)

S3 Fig Portion of 300 ms NOESY spectra for 5´ r(UUGGGC(CAG)3GUCC)2. NOESY spectra showing NH-NH NOEs at 298K. Some of the peaks are overlapped.
(TIF)

S4 Fig Portion of 300 ms NOESY spectra for 5´ r(UUGGGC(CAG)3GUCC)2. NOESY spectra showing base to sugar 1´ correlation at 298K showing. Some of the peaks are overlapped.
(TIF)

S5 Fig Potential energy analysis of RNA model during rMD simulation. (TIF)

S1 Table Data collection and refinement statistics. (DOCX)

S2 Table Sugar and backbone torsional angles (º) calculated for 5´ r(UUGGGC(CAG)_3_GUCC)_2_. (DOCX)

S3 Table Sugar and backbone torsional angles (º) calculated for 5´ r(CCGCAGCGG)_2_. (DOCX)

S4 Table Distances (Å) and angle (º) of atoms for different base pairs of 5´ r(UUGGGC(CAG)_3_GUCC)_2_. (DOCX)

S5 Table Distances (Å) and angle (º) of atoms for different base pairs of 5´ r(CCGCAGCGG)_2_. (DOCX)

S6 Table Global helical parameters calculated for the base pairs of 5´ r(UUGGGC(CAG)3GUCC)_2_. (DOCX)

S7 Table Global helical parameters calculated for the base pairs of 5´ r(CCGCAGCGG)_2_. (DOCX)

S8 Table Helical parameters for different base pairs and steps 5´ r(UUGGGC(CAG)3GUCC)_2_. (DOCX)

S9 Table Helical parameters for different base pairs and steps of 5´ r(CCGCAGCGG)_2_. (DOCX)

S10 Table Helical parameters for different base pairs and steps of 5´ r(UUGGGC(CAG)_3_GUCC)_2_. (DOCX)

S11 Table Helical parameters for different base pairs and steps of 5´ r(CCGCAGCGG)_2_. (DOCX)

S12 Table Major groove widths according to direct P-P distances for the direction of sugar—phosphate backbone in 5´ r(UUGGGC(CAG)_3_GUCC)_2_ structures and their corresponding distances for RNA AU and CG pairs and the distances in B-form DNA. (DOCX)

S13 Table Major groove widths according to direct P-P distances for the direction of sugar phosphate backbone in 5´ r(CCGCAGCGG)_2_ structures. (DOCX)

S14 Table Energy profile of RNA model in rMD simulations (in kcal/mol). (DOCX)
