# Supplementary figures and images for: Structural Insights Reveal the Dynamics of the Repeating r(CAG) Transcript Found in Huntington’s Disease (HD) and Spinocerebellar Ataxias (SCAs)
Source: PLoS One. 2015 Jul 6;10(7):e0131788. doi: 10.1371/journal.pone.0131788 (PMC4493008; doi:10.1371/journal.pone.0131788)

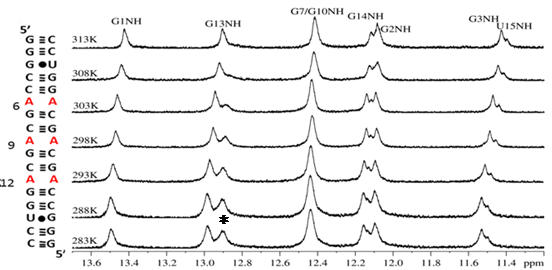

Supplement: S1 Fig — One dimensional proton spectra for 5´ r(UUGGGC(CAG)3GUCC)2 showing imino proton region at variable temperature. Asterisk mark attributes to the appearance of new resonance due to the dynamics in adenine pairs. (TIF) [file pone.0131788.s001.tif]

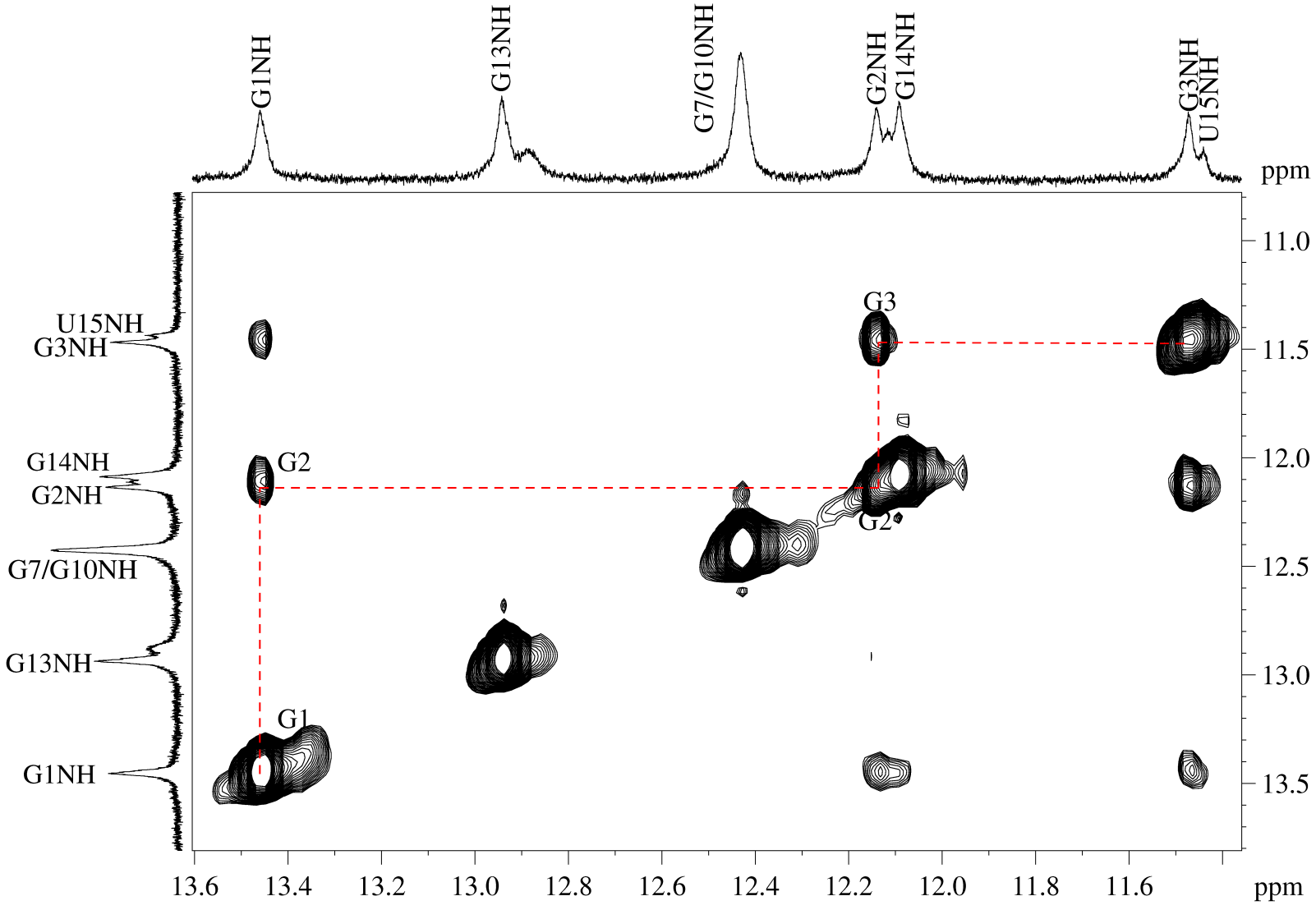

Supplement: S3 Fig — NOESY spectra showing NH-NH NOEs at 298K. Some of the peaks are overlapped. (TIF) [file pone.0131788.s003.tif]

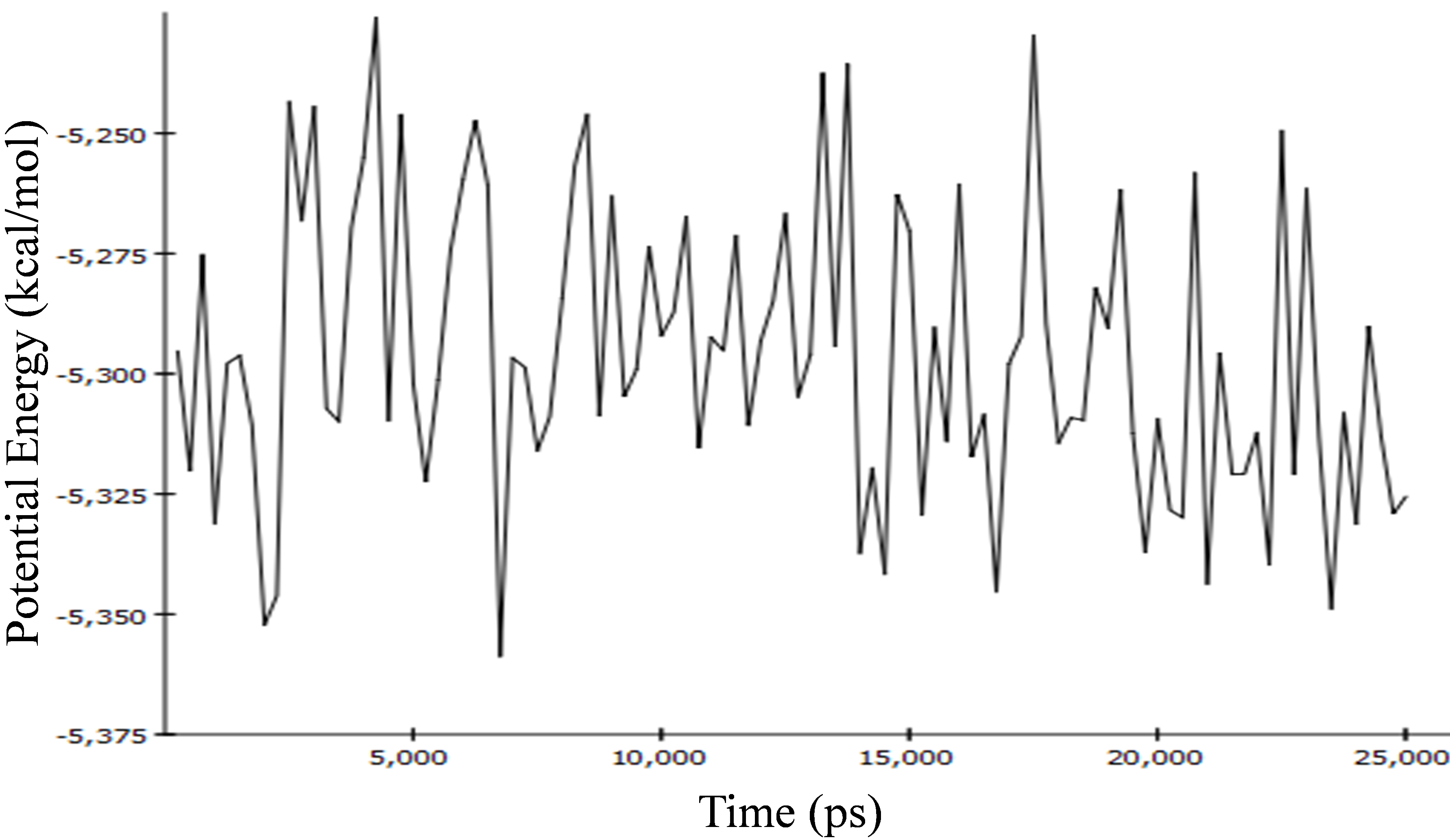

Supplement: S5 Fig — (TIF) [file pone.0131788.s005.tif]
