# Supplementary material for: Structural Insights Reveal the Dynamics of the Repeating r(CAG) Transcript Found in Huntington’s Disease (HD) and Spinocerebellar Ataxias (SCAs)
Source: PLoS One. 2015 Jul 6;10(7):e0131788. doi: 10.1371/journal.pone.0131788 (PMC4493008; doi:10.1371/journal.pone.0131788)
Supplement: S1 Table — (DOCX) [file pone.0131788.s006.docx]

| **S1 Table.** Data collection and refinement statistics. | |
| --- | --- |
|  | r(UUGGGC(C**A**G)_3_GUCC)_2_ |
| **Data collection** |  |
| Space group | H3 |
| Unit cell dimensions |  |
| *a*, *b*, *c* (Å) | 46.0, 46.0, 133.2 |
| *α β γ* (°) | 90, 90, 120 |
| Resolution (Å) | 50-1.95 (2.02-1.95) ^a^ |
| Total measured reflections | 117,578 |
| Unique reflections | 7,642 |
| *R*_merge_ [%]^b^ | 7.0 (45.6) ^a^ |
| *I* /**σ**(*I)* | 21.3 (2.2) ^a^ |
| Completeness (%) | 99.4 (96.8) ^a^ |
| Redundancy | 3.1(3.1) ^a^ |
|  |  |
| **Refinement** |  |
| Resolution (Å) | 44.40-2.30(2.02-2.30) ^a^ |
| Number of reflections | 4,664 |
| *R*_work_ ^c^ / *R*_free_ ^d^ | 21.7/26.1 |
| Number of atoms |  |
| RNA | 816 |
| Ligand/ion | 8 |
| Solvent | 99 |
| Average *B*-factors |  |
| RNA | 41.1 |
| Ligand/ion | 39 |
| Solvent | 53 |
| R.m.s. deviations from ideal geometry |  |
| Bond lengths (Å) | 0.020 |
| Bond angles (°) | 0.627 |

^a^ Values in parentheses are for the highest resolution shell.

^b^ *R*_merge_=∑_h_∑_l_|*I*(h)_l_–<*I*(h)>| / ∑_h_∑_l_*I*(h)_l_, where *I*(h)_l_ is the l th observation of the reflection h and <*I*(h)> is the weighted average intensity for all observations l of reflection h.

^c^ *R*_work_=∑_h_||*F*_obs_(h)|–|*F*_cal_(h)|| /∑_h_|*F*_obs_(h)|, where *F*_obs_(h) and *F*_cal_(h) are the observed and calculated structure factors for reflection h respectively.

^d^ *R*_free_ was calculated as *R*_work_ using the 5% of reflections which were selected randomly and omitted from refinement.
