# Supplementary material for: Structural Insights Reveal the Dynamics of the Repeating r(CAG) Transcript Found in Huntington’s Disease (HD) and Spinocerebellar Ataxias (SCAs)
Source: PLoS One. 2015 Jul 6;10(7):e0131788. doi: 10.1371/journal.pone.0131788 (PMC4493008; doi:10.1371/journal.pone.0131788)
Supplement: S2 Table — (DOCX) [file pone.0131788.s007.docx]

| **S2 Table.** Sugar and backbone torsional angles^a^ (º) calculated for 5´ r(UUGGGC(C**A**G)_3_GUCC)_2_ | | | | | | | |
| --- | --- | --- | --- | --- | --- | --- | --- |
| **Strand I** | | | | | | | |
| **Base** | **α** | **β** | **γ** | **δ** | **ε** | **ξ** | **χ** |
| **G-3** | --- | 166.8 | 33.8 | 83.2 | -136.3 | -73.8 | 179.7 |
| **G-4** | -59.3 | 171.1 | 53.8 | 78.1 | -151.9 | -78.8 | -170.1 |
| **G-5** | -68.8 | -178.8 | 54.2 | 79.3 | -145.1 | -67.4 | -166.4 |
| **C-6** | -65.8 | 173.9 | 52.1 | 76.6 | -152.3 | -67.7 | -160.5 |
| **C-7** | -68.2 | 177.5 | 59.2 | 76.0 | -155.6 | -68.8 | -160.3 |
| **A-8** | -66.6 | 176.2 | 56.2 | 81.0 | -158.9 | -71.9 | -159.5 |
| **G-9** | -75.0 | 175.4 | 65.1 | 79.7 | -143.8 | -106.2 | -163.2 |
| **C-10** | 6.7.0 | 142.3 | 10.8 | 81.7 | -161.0 | -70.0 | -139.8 |
| **A-11** | -86.0 | -176.0 | 55.7 | 80.9 | -164.7 | -71.6 | -153.8 |
| **G-12** | 94.5 | -164.6 | -104.0 | 87.5 | -142.4 | -58.2 | -169.1 |
| **C-13** | -66.6 | 177.3 | 46.2 | 82.6 | -139.5 | -41.9 | -155.7 |
| **A-14** | -129.4 | 106.5 | 156.9 | 82.7 | -118.4 | -81.6 | -0.5 |
| **G-15** | 84.2 | -151.7 | -124.7 | 92.0 | -137.7 | -73.8 | -175.0 |
| **G-16** | -74.3 | -177.8 | 58.8 | 81.9 | -140.7 | -67.4 | -166.3 |
| **U-17** | -69.0 | 174.0 | 52.8 | 78.1 | -152.5 | -80.6 | -154.7 |
| **C-18** | -65.6 | 164.5 | 61.3 | 80.8 | -150.6 | -70.7 | -160.0 |
| **C-19** | -72.5 | -177.2 | 53.8 | 76.1 | --- | --- | -156.0 |
| **Strand II** | | | | | | | |
| **Base** | **α** | **β** | **γ** | **δ** | **ε** | **ξ** | **Χ** |
| **C-19** | -72.8 | -178.2 | 54.0 | 77.0 | --- | --- | -163.4 |
| **C-18** | -61.8 | 161.2 | 58.4 | 82.2 | -152.7 | -71.5 | -157.1 |
| **U-17** | -58.1 | 170.7 | 49.3 | 80.0 | -150.9 | -83.9 | -156.0 |
| **G-16** | -63.5 | 178.8 | 48.3 | 80.2 | -139.5 | -72.9 | -163.4 |
| **G-15** | 149.2 | 176.7 | -168.6 | 84.5 | -137.3 | -80.6 | -175.8 |
| **A-14** | -59.5 | 161.3 | 49.3 | 80.6 | 178.6 | -59.5 | -156.6 |
| **C-13** | -66.7 | 174.1 | 55.4 | 83.1 | -132.9 | -84.8 | -154.3 |
| **G-12** | 149.1 | -153.3 | -179.1 | 83.4 | -131.0 | -79.3 | -179.9 |
| **A-11** | -59.6 | -173.1 | 36.3 | 81.1 | -176.3 | -75.1 | -143.4 |
| **C-10** | -53.0 | 172.3 | 43.3 | 81.0 | -155.3 | -69.4 | -150.0 |
| **G-9** | -61.7 | -176.8 | 51.2 | 80.9 | -154.4 | -73.9 | -155.9 |
| **A-8** | -65.7 | 171.6 | 57.3 | 79.7 | -160.9 | -71.1 | -161.9 |
| **C-7** | -73.5 | 179.4 | 63.0 | 77.1 | -153.2 | -71.9 | -157.8 |
| **C-6** | -62.1 | 169.7 | 54.4 | 75.8 | -150.1 | -67.2 | -164.3 |
| **G-5** | -62.8 | -175.9 | 42.1 | 77.6 | -146.2 | -69.2 | -162.2 |
| **G-4** | -64.1 | 166.4 | 60.3 | 78.9 | -152.4 | -75.4 | -168.3 |
| **G-3** | --- | 167.3 | 36.5 | 85.5 | -136.5 | -76.0 | -175.6 |

**^α^**P **^α^**O5’ **^β^**C5’ **^γ^**C4’ **^δ^**C3’ **^ε^**O3’ **^ξ^**P
