# Supplementary material for: Structural Insights Reveal the Dynamics of the Repeating r(CAG) Transcript Found in Huntington’s Disease (HD) and Spinocerebellar Ataxias (SCAs)
Source: PLoS One. 2015 Jul 6;10(7):e0131788. doi: 10.1371/journal.pone.0131788 (PMC4493008; doi:10.1371/journal.pone.0131788)
Supplement: S3 Table — (DOCX) [file pone.0131788.s008.docx]

| **S3 Table.** Sugar and backbone torsional angles^a^ (º) calculated for 5´ r(CCGC**A**GCGG)_2_ | | | | | | | |
| --- | --- | --- | --- | --- | --- | --- | --- |
| **Strand I** | | | | | | | |
| **Base** | **α** | **β** | **γ** | **δ** | **ε** | **ξ** | **χ** |
| **C-1** | --- | 165.3 | 64.5 | 73.2 | -154.5 | -67.9 | -160.0 |
| **C-2** | -71.4 | 168.5 | 61.4 | 75.6 | -159.7 | -69.2 | -160.9 |
| **G-3** | -70.4 | 171.4 | 56.3 | 77.2 | -158.3 | -67.0 | -158.8 |
| **C-4** | -65.3 | 166.9 | 56.2 | 79.8 | -119.6 | -101.8 | -164.9 |
| **A-5** | -59.9 | 102.8 | 85.4 | 71.3 | -125.7 | -74.8 | 179.4 |
| **G-6** | -76.9 | -174.6 | 57.7 | 80.0 | -157.1 | -67.2 | -166.4 |
| **C-7** | -76.2 | 174.9 | 63.8 | 77.6 | -158.8 | -66.6 | -149.8 |
| **G-8** | -70.1 | 170.9 | 60.5 | 75.1 | -159.3 | -67.4 | -163.6 |
| **G-9** | -65.3 | 172.1 | 53.3 | 85.7 | --- | --- | -162.2 |
| **Strand II** | | | | | | | |
| **Base** | **α** | **β** | **γ** | **δ** | **ε** | **ξ** | **χ** |
| **C-1** | -61.1 | 165.5 | 60.6 | 76.5 | --- | --- | -162.5 |
| **C-2** | -56.8 | 163.2 | 52.0 | 79.1 | -151.7 | -74.6 | -163.1 |
| **G-3** | -65.2 | 163.4 | 58.8 | 76.4 | -155.5 | -74.1 | -154.8 |
| **C-4** | -63.7 | 177.0 | 54.0 | 80.4 | -154.7 | -72.0 | -170.0 |
| **A-5** | -50.6 | 127.6 | 56.0 | 70.1 | -159.4 | -68.3 | -165.3 |
| **G-6** | -65.6 | 162.1 | 61.5 | 80.1 | -145.8 | -90.1 | -164.0 |
| **C-7** | -56.2 | 162.7 | 51.0 | 77.3 | -152.9 | -69.2 | -156.9 |
| **G-8** | -59.6 | 164.8 | 55.7 | 74.1 | -157.0 | -70.0 | -155.5 |
| **G-9** | --- | 169.6 | 54.5 | 77.4 | -152.8 | -74.2 | -165.0 |

^a^P **^α^**O5’ **^β^**C5’ **^γ^**C4’ **^δ^**C3’ **^ε^**O3’ **^ξ^**P
