# Supplementary material for: Structural Insights Reveal the Dynamics of the Repeating r(CAG) Transcript Found in Huntington’s Disease (HD) and Spinocerebellar Ataxias (SCAs)
Source: PLoS One. 2015 Jul 6;10(7):e0131788. doi: 10.1371/journal.pone.0131788 (PMC4493008; doi:10.1371/journal.pone.0131788)
Supplement: S4 Table — (DOCX) [file pone.0131788.s009.docx]

| **S4 Table.** Distances (Å) and angle (º) of atoms for different base pairs of  5´ r(UUGGGC(C**A**G)_3_GUCC)_2_ | | | | | |
| --- | --- | --- | --- | --- | --- |
| **Base Pair** | **λ (I)^b^(º)** | **λ (II)^b^(º)** | **C1'-C1'(Å)** | **RN9-YN1(Å)** | **RC8-YC6(Å)** |
| **G3-C19** | 49.9 | 55.5 | 10.8 | 9.0 | 9.9 |
| **G4-C18** | 55.8 | 59.0 | 10.5 | 8.9 | 9.9 |
| **G5-U17** | 43.5 | 69.2 | 10.5 | 8.9 | 9.8 |
| **C6-G16** | 58.0 | 54.0 | 10.7 | 9.0 | 10.0 |
| **C7-G15** | 55.5 | 53.3 | 10.6 | 8.9 | 9.9 |
| **A8 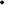 A14** | 69.5 | 65.6 | 11.3 | 10.2 | 11.5 |
| **G9-C13** | 59.0 | 60.1 | 10.3 | 8.8 | 9.9 |
| **C10-G12** | 52.9 | 54.1 | 10.8 | 9.0 | 9.9 |
| **A11 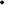 A11** | 61.5 | 65.5 | 11.7 | 10.4 | 11.6 |
| **G12-C10** | 41.6 | 56.6 | 10.5 | 8.5 | 9.3 |
| **C13-G9** | 62.5 | 57.4 | 10.3 | 8.8 | 9.9 |
| **A14 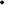 A8** | 35.2 | 64.5 | 11.1 | 9.3 | 8.9 |
| **G15-C7** | 53.4 | 56.8 | 10.6 | 8.9 | 9.9 |
| **G16-C6** | 52.5 | 55.9 | 10.7 | 9.0 | 9.9 |
| **U17-G5** | 69.7 | 44.0 | 10.4 | 8.8 | 9.8 |
| **C18-G4** | 58.3 | 56.0 | 10.5 | 8.9 | 10.0 |
| **C19-G3** | 56.0 | 52.9 | 10.6 | 8.9 | 9.8 |

^b^Lambda is the virtual angle between C1'-YN1 or C1'-RN9 glycosidic bonds and the base-pair C1'-C1' line.

C1'-C1' is the distance between C1' atoms for each base-pair.

RN9-YN1 is the distance between RN9-YN1 atoms for each base-pair.

RC8-YC6 is the distance between RC8-YC6 atoms for each base-pair.
