# Supplementary material for: Structural Insights Reveal the Dynamics of the Repeating r(CAG) Transcript Found in Huntington’s Disease (HD) and Spinocerebellar Ataxias (SCAs)
Source: PLoS One. 2015 Jul 6;10(7):e0131788. doi: 10.1371/journal.pone.0131788 (PMC4493008; doi:10.1371/journal.pone.0131788)
Supplement: S5 Table — (DOCX) [file pone.0131788.s010.docx]

| **S5 Table.**  Distances (Å) and angle (º) of atoms for different base pairs of 5’ r(CCGC**A**GCGG)_2_ | | | | | |
| --- | --- | --- | --- | --- | --- |
| **Base Pair** | **λ (I)^b^(º)** | **λ (II)^b^(º)** | **C1'-C1'(Å)** | **RN9-YN1(Å)** | **RC8-YC6(Å)** |
| **C1-G9** | 55.5 | 55.7 | 10.8 | 9.1 | 9.9 |
| **C2-G8** | 55.6 | 55.9 | 10.7 | 9.0 | 9.8 |
| **G3-C7** | 55.2 | 53.3 | 10.8 | 9.0 | 9.8 |
| **C4-G6** | 54.5 | 57.3 | 10.7 | 9.1 | 9.8 |
| **A5 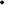 A5** | 43.9 | 58.1 | **12.7** | 10.8 | 11.3 |
| **G6-C4** | 56.1 | 53.6 | 10.8 | 9.1 | 9.9 |
| **C7-G3** | 50.2 | 54.6 | 10.8 | 9.0 | 9.8 |
| **G8-C2** | 55.3 | 55.4 | 10.6 | 9.0 | 9.8 |
| **G9-C1** | 55.4 | 54.5 | 10.7 | 9.0 | 9.9 |

^b^Lambda is the virtual angle between C1'-YN1 or C1'-RN9 glycosidic bonds and the base-pair C1'-C1' line.

C1'-C1' is the distance between C1' atoms for each base-pair.

RN9-YN1 is the distance between RN9-YN1 atoms for each base-pair.

RC8-YC6 is the distance between RC8-YC6 atoms for each base-pair.
