# Supplementary material for: Structural Insights Reveal the Dynamics of the Repeating r(CAG) Transcript Found in Huntington’s Disease (HD) and Spinocerebellar Ataxias (SCAs)
Source: PLoS One. 2015 Jul 6;10(7):e0131788. doi: 10.1371/journal.pone.0131788 (PMC4493008; doi:10.1371/journal.pone.0131788)
Supplement: S6 Table — (DOCX) [file pone.0131788.s011.docx]

| **S6 Table.** Global helical parameters calculated for the base pairs of 5´ r(UUGGGC(C**A**G)_3_GUCC)_2_ | | | | |
| --- | --- | --- | --- | --- |
| **Base pair** | **Displacement**  **(Å)** | **Angle**  **(º)** | **Twist**  **(º)** | **Rise**  **(Å)** |
| **G3-C19** | 7.72 | 13.90 | 33.18 | 2.36 |
| **G4-C18** | 7.58 | 13.66 | 37.24 | 2.65 |
| **G5-U17** | 8.04 | 13.15 | 25.75 | 2.90 |
| **C6-G16** | 7.74 | 12.51 | 34.37 | 2.47 |
| **C7-G15** | 6.49 | 14.92 | 24.45 | 2.14 |
| **A8 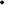 A14** | 6.69 | 16.80 | 30.83 | 2.74 |
| **G9-C13** | 7.39 | 11.16 | 37.51 | 2.57 |
| **C10-G12** | 7.64 | 11.43 | 26.76 | 2.90 |
| **A11 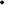 A11** | 8.38 | 15.08 | 25.99 | 2.64 |
| **G12-C10** | 7.75 | 10.87 | 33.28 | 2.61 |
| **C13-G9** | 7.38 | 12.66 | 29.80 | 2.59 |
| **A14 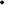 A8** | 6.49 | 16.07 | 27.58 | 2.18 |
| **G15-C7** | 6.58 | 14.25 | 34.65 | 2.49 |
| **G16-C6** | 7.77 | 11.18 | 26.36 | 2.85 |
| **U17-G5** | 8.08 | 12.30 | 37.56 | 2.55 |
| **C18-G4** | 7.47 | 14.44 | 32.67 | 2.55 |
| **C19-G3** | 7.43 | 15.96 | --- | --- |
