# Supplementary material for: Structural Insights Reveal the Dynamics of the Repeating r(CAG) Transcript Found in Huntington’s Disease (HD) and Spinocerebellar Ataxias (SCAs)
Source: PLoS One. 2015 Jul 6;10(7):e0131788. doi: 10.1371/journal.pone.0131788 (PMC4493008; doi:10.1371/journal.pone.0131788)
Supplement: S7 Table — (DOCX) [file pone.0131788.s012.docx]

| **S7 Table.** Global helical parameters calculated for the base pairs of 5´ r(CCGC**A**GCGG)_2_ | | | | |
| --- | --- | --- | --- | --- |
| **Base pair** | **Displacement**  **(Å)** | **Angle**  **(º)** | **Twist**  **(º)** | **Rise**  **(Å)** |
| **C1-G9** | 6.33 | 15.37 | 32.81 | 2.80 |
| **C2-G8** | 6.17 | 14.49 | 32.98 | 2.76 |
| **G3-C7** | 6.18 | 14.55 | 32.51 | 2.90 |
| **C4-G6** | 6.25 | 12.97 | 30.54 | 2.92 |
| **A5 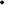 A5** | 5.97 | 13.38 | 34.89 | 2.73 |
| **G6-C4** | 6.28 | 13.26 | 31.53 | 2.95 |
| **C7-G3** | 6.31 | 14.77 | 34.09 | 2.77 |
| **G8-C2** | 6.30 | 14.80 | 32.67 | 2.81 |
| **G9-C1** | 6.40 | 15.12 | --- | --- |
