# Supplementary material for: Structural Insights Reveal the Dynamics of the Repeating r(CAG) Transcript Found in Huntington’s Disease (HD) and Spinocerebellar Ataxias (SCAs)
Source: PLoS One. 2015 Jul 6;10(7):e0131788. doi: 10.1371/journal.pone.0131788 (PMC4493008; doi:10.1371/journal.pone.0131788)
Supplement: S8 Table — (DOCX) [file pone.0131788.s013.docx]

| **S8 Table.** Helical parameters for different base pairs and steps of  5´ r(UUGGGC(C**A**G)_3_GUCC)_2_ | | | | | | |
| --- | --- | --- | --- | --- | --- | --- |
|  | **Local base-pair parameters** | | | | | |
| **Base Pair** | **Shear**  **(Å)** | **Stretch**  **(Å)** | **Stagger**  **(Å)** | **Buckle**  **(º)** | **Propeller**  **(º)** | **Opening**  **(º)** |
| **G3-C19** | -0.27 | -0.12 | -0.15 | -2.53 | 3.07 | 0.23 |
| **G4-C18** | -0.39 | -0.06 | -0.38 | -9.63 | -13.05 | 4.70 |
| **G5-U17** | -2.36 | -0.49 | -0.17 | -5.10 | -7.54 | 0.42 |
| **C6-G16** | 0.46 | -0.07 | -0.26 | 3.22 | -10.47 | 0.28 |
| **C7-G15** | 0.24 | -0.16 | -0.10 | 1.09 | -11.47 | 1.50 |
| **A8 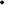 A14** | 0.54 | 1.98 | 0.42 | 4.02 | -9.67 | 26.10 |
| **G9-C13** | -0.12 | -0.12 | -0.25 | 0.88 | -21.77 | 8.60 |
| **C10-G12** | 0.08 | -0.10 | -0.21 | 7.60 | -21.49 | 0.46 |
| **A11 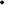 A11** | -0.43 | 1.95 | -0.05 | -1.82 | -6.13 | 17.48 |
| **G12-C10** | -1.39 | -0.80 | 0.19 | -5.49 | -19.64 | -8.06 |
| **C13-G9** | 0.37 | -0.09 | 0.41 | -2.60 | -17.98 | 7.22 |
| **A14 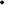 A5** | -1.72 | -3.69 | -0.72 | 7.20 | 10.14 | 80.77 |
| **G15-C7** | -0.21 | -0.19 | -0.05 | -3.46 | -15.22 | 1.59 |
| **G16-C6** | -0.49 | -0.19 | -0.04 | -3.30 | -12.58 | -2.33 |
| **U17-G5** | 2.31 | -0.54 | -0.45 | 3.35 | -10.19 | 0.80 |
| **C18-G4** | 0.27 | -0.05 | -0.13 | 4.03 | -11.67 | 4.63 |
| **C19-G3** | 0.26 | -0.22 | 0.03 | 5.52 | -3.91 | 0.81 |
| **Average** | -0.17 | -0.17 | -0.11 | 0.18 | -10.56 | 8.54 |
| **Std.Dev.** | 1.03 | 1.18 | 0.28 | 4.90 | 8.31 | 20.18 |
