# Supplementary material for: Structural Insights Reveal the Dynamics of the Repeating r(CAG) Transcript Found in Huntington’s Disease (HD) and Spinocerebellar Ataxias (SCAs)
Source: PLoS One. 2015 Jul 6;10(7):e0131788. doi: 10.1371/journal.pone.0131788 (PMC4493008; doi:10.1371/journal.pone.0131788)
Supplement: S9 Table — (DOCX) [file pone.0131788.s014.docx]

| **S9 Table.**  Helical parameters for different base pairs and steps of 5´ r(CCGC**A**GCGG)_2_ | | | | | | |
| --- | --- | --- | --- | --- | --- | --- |
|  | **Local base-pair parameters** | | | | | |
| **Base pair** | **Shear**  **(Å)** | **Stretch**  **(Å)** | **Stagger**  **(Å)** | **Buckle**  **(º)** | **Propeller**  **(º)** | **Opening**  **(º)** |
| **C1-G9** | 0.32 | -0.11 | -0.25 | 0.06 | -1.49 | -0.89 |
| **C2-G8** | 0.06 | -0.20 | -0.53 | 1.51 | -1.93 | -3.15 |
| **G3-C7** | -0.24 | -0.19 | -0.13 | -1.79 | -1.22 | -2.07 |
| **C4-G6** | 0.11 | -0.16 | 0.05 | 4.93 | -0.54 | -1.11 |
| **A5 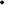 A5** | -1.61 | 1.44 | 0.29 | -0.34 | -11.28 | -14.10 |
| **G6-C4** | -0.26 | -0.16 | -0.10 | -0.04 | -0.23 | -1.97 |
| **C7-G3** | 0.21 | -0.18 | -0.52 | 3.69 | -3.22 | 0.66 |
| **G8-C2** | 0.01 | -0.15 | -0.50 | -5.60 | -3.89 | -1.74 |
| **G9-C1** | -0.14 | -0.11 | -0.17 | -0.52 | -2.18 | 0.70 |
| **Average** | -0.17 | 0.02 | -0.21 | 0.21 | -2.89 | -2.63 |
| **Std. Dev.** | 0.57 | 0.53 | 0.28 | 3.06 | 3.36 | 4.48 |
