# Supplementary material for: Structural Insights Reveal the Dynamics of the Repeating r(CAG) Transcript Found in Huntington’s Disease (HD) and Spinocerebellar Ataxias (SCAs)
Source: PLoS One. 2015 Jul 6;10(7):e0131788. doi: 10.1371/journal.pone.0131788 (PMC4493008; doi:10.1371/journal.pone.0131788)
Supplement: S10 Table — (DOCX) [file pone.0131788.s015.docx]

| **S10 Table.** Helical parameters for different base pairs and steps of 5´ r(UUGGGC(C**A**G)_3_GUCC)_2_ | | | | | | | | | | | | |
| --- | --- | --- | --- | --- | --- | --- | --- | --- | --- | --- | --- | --- |
| **Local base-pair step parameters** | | | | | | | **Local base-pair helical parameters** | | | | | |
| **Step** | **Shift (Å)** | **Slide (Å)** | **Rise**  **(Å)** | **Tilt**  **(º)** | **Roll**  **(º)** | **Twist**  **(º)** | **X-disp**  **(Å)** | **Y-disp**  **(Å)** | **h-Rise**  **(Å)** | **Incl.**  **(º)** | **Tip**  **(º)** | **h-Twist**  **(º)** |
| **GG/CC** | 0.37 | -1.97 | 3.56 | 2.63 | 5.15 | 31.35 | -4.56 | -0.18 | 3.23 | 9.43 | -4.82 | 31.86 |
| **GG/UC** | -0.49 | -1.66 | 2.95 | -5.05 | 9.47 | 24.45 | -5.65 | -0.02 | 2.22 | 21.11 | 11.26 | 26.67 |
| **GC/GU** | -0.05 | -1.34 | 3.07 | -1.19 | 9.21 | 40.21 | -2.79 | -0.05 | 2.71 | 13.18 | 1.71 | 41.22 |
| **CC/GG** | 0.55 | -1.93 | 3.39 | 1.23 | 7.27 | 31.99 | -4.64 | -0.78 | 2.91 | 12.97 | -2.20 | 32.81 |
| **CA/AG** | 0.90 | -2.22 | 3.06 | 0.36 | 4.33 | 24.67 | -6.25 | -1.98 | 2.65 | 10.03 | -0.84 | 25.04 |
| **AG/CA** | -0.93 | -1.34 | 3.02 | 1.38 | 14.87 | 25.56 | -5.19 | 2.07 | 1.91 | 30.51 | -2.82 | 29.54 |
| **GC/GC** | -0.26 | -1.34 | 3.13 | -0.69 | 5.69 | 37.19 | -2.76 | 0.31 | 2.91 | 8.86 | 1.08 | 37.61 |
| **CA/AG** | 0.08 | -1.21 | 3.45 | -0.70 | 14.20 | 23.14 | -6.02 | -0.34 | 2.33 | 31.83 | 1.58 | 27.10 |
| **AG/CA** | -0.50 | -2.30 | 3.46 | -5.21 | 7.92 | 19.61 | -9.02 | -0.62 | 2.41 | 21.69 | 14.25 | 21.76 |
| **GC/GC** | 0.95 | -0.95 | 3.04 | -0.25 | 5.26 | 41.79 | -1.82 | -1.34 | 2.90 | 7.34 | 0.35 | 42.11 |
| **CA/AG** | 0.83 | 3.06 | -1.73 | 167.62 | -37.12 | -140.13 | -1.59 | 0.12 | -1.68 | 18.61 | 84.05 | -177.17 |
| **AG/CA** | 0.68 | -4.16 | -1.61 | 135.09 | -107.46 | 143.46 | -2.19 | -0.47 | -0.55 | -53.85 | -67.69 | 177.69 |
| **GG/CC** | -0.70 | -2.01 | 3.22 | -3.98 | 7.86 | 31.60 | -4.81 | 0.61 | 2.72 | 14.10 | 7.14 | 32.77 |
| **GU/GC** | 0.04 | -1.30 | 3.16 | 4.11 | 6.89 | 41.03 | -2.49 | 0.35 | 2.91 | 9.71 | -5.79 | 41.78 |
| **UC/GG** | 0.53 | -1.82 | 2.99 | 3.11 | 10.86 | 24.25 | -6.23 | -0.50 | 2.05 | 24.24 | -6.95 | 26.71 |
| **CC/GG** | -0.24 | -1.81 | 3.35 | 0.15 | 5.74 | 31.34 | -4.32 | 0.46 | 2.98 | 10.51 | -0.28 | 31.85 |
| **Average** | 0.11 | -1.52 | 2.59 | 18.66 | -1.87 | 26.97 | -4.40 | -0.15 | 2.16 | 11.89 | 1.88 | 28.09 |
| **Std. Dev.** | 0.60 | 1.42 | 1.68 | 52.21 | 30.49 | 53.14 | 2.03 | 0.90 | 1.35 | 19.14 | 28.40 | 65.97 |
