# Supplementary material for: Structural Insights Reveal the Dynamics of the Repeating r(CAG) Transcript Found in Huntington’s Disease (HD) and Spinocerebellar Ataxias (SCAs)
Source: PLoS One. 2015 Jul 6;10(7):e0131788. doi: 10.1371/journal.pone.0131788 (PMC4493008; doi:10.1371/journal.pone.0131788)
Supplement: S11 Table — (DOCX) [file pone.0131788.s016.docx]

| **S11 Table.** Helical parameters for different base pairs and steps of 5´ r(CCGC**A**GCGG)_2_ | | | | | | | | | | | | |
| --- | --- | --- | --- | --- | --- | --- | --- | --- | --- | --- | --- | --- |
| **Local base-pair step parameters** | | | | | | | **Local base-pair helical parameters** | | | | | |
| **Step** | **Shift (Å)** | **Slide (Å)** | **Rise**  **(Å)** | **Tilt**  **(º)** | **Roll**  **(º)** | **Twist**  **(º)** | **X-disp**  **(Å)** | **Y-disp**  **(Å)** | **h-Rise**  **(Å)** | **Incl.**  **(º)** | **Tip**  **(º)** | **h-Twist**  **(º)** |
| **CC/GG** | -0.06 | -1.34 | 3.27 | 3.26 | 9.66 | 29.7 | -4.18 | 0.69 | 2.69 | 18.19 | -6.13 | 31.36 |
| **CG/CG** | 0.08 | -1.34 | 3.45 | -2.38 | 11.21 | 29.36 | -4.52 | -0.59 | 2.75 | 21.12 | 4.48 | 31.47 |
| **GC/GC** | 0.03 | -1.45 | 3.11 | -1.58 | 7.56 | 33.95 | -3.47 | -0.26 | 2.73 | 12.75 | 2.66 | 34.8 |
| **CA/AG** | -0.25 | -1.47 | 3.45 | 1.34 | 10.35 | 21.93 | -6.62 | 0.99 | 2.49 | 25.44 | -3.29 | 24.26 |
| **AG/CA** | 0.34 | -1.62 | 3.27 | -1.88 | 8.60 | 38.89 | -3.33 | -0.71 | 2.85 | 12.72 | 2.78 | 39.84 |
| **GC/GC** | 0.04 | -1.32 | 3.23 | 4.48 | 8.53 | 33.86 | -3.38 | 0.56 | 2.81 | 14.28 | -7.50 | 35.17 |
| **CG/CG** | -0.07 | -1.10 | 3.71 | -0.03 | 11.19 | 30.66 | -4.07 | 0.12 | 3.13 | 20.33 | 0.06 | 32.59 |
| **GG/CC** | 0.13 | -1.34 | 3.19 | -2.12 | 9.00 | 29.82 | -4.05 | -0.62 | 2.67 | 16.97 | 3.99 | 31.19 |
| **Average** | 0.03 | -1.37 | 3.33 | 0.14 | 9.51 | 31.02 | -4.2 | 0.02 | 2.76 | 17.73 | -0.37 | 32.59 |
| **Std. Dev.** | 0.17 | 0.15 | 0.19 | 2.63 | 1.32 | 4.89 | 1.07 | 0.66 | 0.18 | 4.48 | 4.70 | 4.44 |
