# Supplementary material for: Structural Insights Reveal the Dynamics of the Repeating r(CAG) Transcript Found in Huntington’s Disease (HD) and Spinocerebellar Ataxias (SCAs)
Source: PLoS One. 2015 Jul 6;10(7):e0131788. doi: 10.1371/journal.pone.0131788 (PMC4493008; doi:10.1371/journal.pone.0131788)
Supplement: S12 Table — (DOCX) [file pone.0131788.s017.docx]

| **S12 Table.** Major groove widths according to direct P-P distances for the direction of sugar-phosphate backbone in 5´ r(UUGGGC(C**A**G)_3_GUCC)_2_ structures and their corresponding distances for RNA AU and CG pairs and the distances in B-form DNA. | | | | |
| --- | --- | --- | --- | --- |
| Step | Major Groove ( Å) | | | |
|  | RNA | | | DNA |
|  | CAG | CG Pair | AU pair | B-DNA |
| **GG/CC** | --- | --- | --- | --- |
| **GG/UC** | --- | --- | --- | --- |
| **GC/GU** | 17.4 | 9.1 | 9.1 | 11.4 |
| **CC/GG** | 17.1 | 9.1 | 9.1 | 11.4 |
| **CA/AG** | 16.7 | 9.1 | 9.1 | 11.4 |
| **AG/CA** | 16.9 | 9.1 | 9.1 | 11.4 |
| **GC/GC** | 17.3 | 9.1 | 9.1 | 11.4 |
| **CA/AG** | 17.3 | 9.1 | 9.1 | 11.4 |
| **AG/CA** | 17.2 | 9.1 | 9.1 | 11.4 |
| **GC/GC** | 17.3 | 9.1 | 9.1 | 11.4 |
| **CA/AG** | 16.9 | 9.1 | 9.1 | 11.4 |
| **AG/CA** | 16.6 | 9.1 | 9.1 | 11.4 |
| **GG/CC** | 16.9 | 9.1 | 9.1 | 12.1 |
| **GU/GC** | 17.2 | 9.1 | 9.1 | 11.4 |
| **UC/GG** | --- | --- | --- | --- |
| **CC/GG** | --- | --- | --- | --- |
