# Supplementary material for: Structural Insights Reveal the Dynamics of the Repeating r(CAG) Transcript Found in Huntington’s Disease (HD) and Spinocerebellar Ataxias (SCAs)
Source: PLoS One. 2015 Jul 6;10(7):e0131788. doi: 10.1371/journal.pone.0131788 (PMC4493008; doi:10.1371/journal.pone.0131788)
Supplement: S13 Table — (DOCX) [file pone.0131788.s018.docx]

| **S13 Table.**  Major groove widths according to direct P-P distances for the direction of sugar-phosphate backbone in 5´ r(CCGC**A**GCGG)_2_ structures | | | | |
| --- | --- | --- | --- | --- |
| **Step** | **Minor Groove** | | **Major Groove** | |
|  | **P-P** | **Refined** | **P-P** | **Refined** |
| **CC/GG** | --- | --- | --- | --- |
| **CG/CG** | --- | --- | --- | --- |
| **GC/GC** | 17.9 | --- | 15.7 | --- |
| **CA/AG** | 17.8 | 16.4 | 15.6 | 12.8 |
| **AG/CA** | 17.8 | 16.4 | 15.5 | 12.8 |
| **GC/GC** | 17.9 | --- | 15.3 | --- |
| **CG/CG** | --- | --- | --- | --- |
| **GG/CC** | --- | --- | --- | --- |
