# Supplementary material for: Structural Insights Reveal the Dynamics of the Repeating r(CAG) Transcript Found in Huntington’s Disease (HD) and Spinocerebellar Ataxias (SCAs)
Source: PLoS One. 2015 Jul 6;10(7):e0131788. doi: 10.1371/journal.pone.0131788 (PMC4493008; doi:10.1371/journal.pone.0131788)
Supplement: S14 Table — (DOCX) [file pone.0131788.s019.docx]

| **S14 Table. Energy** profile of RNA model in rMD simulations (in kcal/mol). | | | | | | | | | |
| --- | --- | --- | --- | --- | --- | --- | --- | --- | --- |
| **Total energy** | **Kinetic energy** | **Potential energy** | **Bond energy** | **Angle energy** | **Torsion energy** | **Improper torsion**  **Energy** | **Vander-waal energy** | **Electrostatic energy** | **Harmonic energy** |
| -3,680.40 | 1,678.50 | -5,358.90 | 101.64 | 404.42 | 357.27 | 38.273 | 193.58 | -4,853.70 | 206.70 |
